# Association between variations in the fat mass and obesity-associated gene and pancreatic cancer risk: a case–control study in Japan

**DOI:** 10.1186/1471-2407-13-337

**Published:** 2013-07-08

**Authors:** Yingsong Lin, Junko Ueda, Kiyoko Yagyu, Hiroshi Ishii, Makoto Ueno, Naoto Egawa, Haruhisa Nakao, Mitsuru Mori, Keitaro Matsuo, Shogo Kikuchi

**Affiliations:** 1 Department of Public Health, Aichi Medical University School of Medicine, 1-1 Yazakokarimata, Nagakute, Aichi 480-1195, Japan; 2 Hepatobiliary and Pancreatic Section, Gastroenterological Division, Cancer Institute Hospital, Tokyo, Japan; 3 Hepatobiliary and Pancreatic Medical Oncology Division, Kanagawa Cancer Center Hospital, Kanagawa, Japan; 4 Department of Internal Medicine, Tokyo Metropolitan Matsuzawa Hospital, Tokyo, Japan; 5 Department of Internal Medicine, Tokyo Metropolitan Komagome Hospital, Tokyo, Japan; 6 Division of Gastroenterology, Department of Internal Medicine, Aichi Medical University School of Medicine, Nagakute, Japan; 7 Department of Public Health, Sapporo Medical University School of Medicine, Sapporo, Japan; 8 Department of Preventive Medicine, Kyushu University Faculty of Medical Science, Fukuoka, Japan

**Keywords:** The fat mass and obesity-associated gene, Pancreatic cancer, rs9939609, Case–control study

## Abstract

**Background:**

It is clear that genetic variations in the fat mass and obesity-associated (FTO) gene affect body mass index and the risk of obesity. Given the mounting evidence showing a positive association between obesity and pancreatic cancer, this study aimed to investigate the relation between variants in the FTO gene, obesity and pancreatic cancer risk.

**Methods:**

We conducted a hospital-based case–control study in Japan to investigate whether genetic variations in the FTO gene were associated with pancreatic cancer risk. We genotyped rs9939609 in the FTO gene of 360 cases and 400 control subjects. An unconditional logistic model was used to estimate the odds ratio (OR) and 95% confidence interval (CI) for the association between rs9939609 and pancreatic cancer risk.

**Results:**

The minor allele frequency of rs9939609 was 0.18 among control subjects. BMI was not associated with pancreatic cancer risk. Compared with individuals with the common homozygous TT genotype, those with the heterozygous TA genotype and the minor homozygous AA genotype had a 48% (OR=1.48; 95%CI: 1.07–2.04), and 66% increased risk (OR=1.66; 95%CI: 0.70–3.90), respectively, of pancreatic cancer after adjustment for sex, age, body mass index, cigarette smoking and history of diabetes. The per-allele OR was 1.41 (95%CI: 1.07–1.85). There were no significant interactions between TA/AA genotypes and body mass index.

**Conclusions:**

Our findings indicate that rs9939609 in the FTO gene is associated with pancreatic cancer risk in Japanese subjects, possibly through a mechanism that is independent of obesity. Further investigation and replication of our results is required in other independent samples.

## Background

In 2010, approximately 28,000 Japanese subjects died from pancreatic cancer, making it the fifth leading cause of cancer deaths in Japan [1]. Despite extensive research efforts, the etiology of pancreatic cancer remains poorly understood. Cigarette smoking and long-standing type II diabetes are two well-established risk factors, based on consistent findings from epidemiologic studies [2,3]. In addition, being overweight and obese have been implicated in the development of pancreatic cancer [4], with statistically significant, positive associations observed in large cohort studies conducted in Western countries [5-7], and corroborated in at least four meta-analyses [8-11] and three pooled analyses [12-14]. The positive association between body mass index (BMI) and pancreatic cancer, however, has not been clearly observed in Asian populations. To date, four cohort studies have examined the association between BMI and pancreatic cancer in Asians, but the results have been inconsistent and inconclusive [15-18].

Recently, genome-wide association (GWA) studies have identified at least 30 loci that affect BMI and the risk of obesity [19]. Among these loci, the fat mass and obesity-associated (FTO) gene, which was first identified in a GWA study of diabetes in 2007 [20], has the strongest influence on BMI and obesity. Rs9939609, located in the first intron of the FTO gene, was found to be associated with both BMI and type II diabetes in subsequent GWA studies in diverse populations [21-23]. The association of rs9939609 with various traits, including hip circumference, energy intake and total mortality has also been studied [24-26]. In addition, rs9939609 genotypes have been linked with the risk of prostate, breast and endometrial cancers [27-29]. The association between genetic variations in the FTO gene and the risk of pancreatic cancer, however, is not clear. Of the three studies that examined this association, only one case–control study, conducted at the MD Anderson Cancer Center in the United States, reported that the minor A allele of FTO, rs9939609, was associated with an increased risk of pancreatic cancer among overweight subjects [30]. Another two studies examined rs8050136 of the FTO gene, with one study reporting a positive association [31], and the other no association [32].

Given the mounting evidence showing a positive association between obesity and pancreatic cancer, we hypothesized that variants in the FTO gene may be associated with pancreatic cancer risk through effects on obesity or other mechanisms. In a search of the literature for obesity-related genetic variants, we found that FTO rs9939609 was the most widely studied single nucleotide polymorphism (SNP), and has been found to exert strong effects on BMI, as well as diabetes. Furthermore, it showed strong linkage disequilibrium with other SNPs in the FTO gene, such as rs8050135 and rs17817449 [22]. We therefore investigated the association between FTO rs9939609 and pancreatic cancer risk in a case–control study in Japan.

## Methods

### Study subjects

Our study is an ongoing hospital-based case–control study focusing on the role of genetic polymorphisms and gene-environment interaction in pancreatic cancer. For the present analysis, eligible cases were patients aged older than 20 years, who were newly diagnosed with pancreatic cancer in five hospitals located in central, north and Tokyo metropolitan areas from April 1, 2010 through May 15, 2012. The diagnosis of pancreatic cancer was based on imaging modalities or pathologic reports. The response rate among cases was 85% (441/516) as of July 1, 2012. Almost all of the cases were approached within a week after the diagnosis of pancreatic cancer, and very few cases died before they were invited to participate in our study. During the same period, we recruited control subjects with no diagnosis of cancer from inpatients and outpatients from the participating hospitals where the cases were enrolled, as well as relatives of inpatients, and individuals undergoing a medical checkup in one of the participating hospitals. Control subjects were eligible if they were more than 20 years old and had no prior cancer diagnoses. Recruitment of controls was accomplished by approaching eligible participants in the hospitals who satisfied the study requirements, and the response rate was 98% (525/534). Control subjects had a variety of diseases, such as anemia, gastric ulcer, and irritable bowel syndrome. Control subjects were matched with case patients according to sex and age (within 10-year categories). As a result, data from 360 case patients and 400 control subjects were included in the present analysis.

All subjects provided written, informed consent. This study was approved by the ethical board of Aichi Medical University (Nagakute, Japan), the Institutional Review Board (IRB) of Cancer Institute Hospital (Tokyo, Japan), the IRB of Kanagawa Cancer Center Hospital (Kanagawa, Japan), the IRB of Tokyo Metropolitan Komagome Hospital (Tokyo, Japan), and the IRB of Sapporo Medical University (Sapporo, Japan).

### Data collection

Study subjects were asked to fill out a self-administered questionnaire including information on demographic characteristics, medical history, and lifestyle factors, such as cigarette smoking, alcohol consumption and dietary intake. For body weight, data on usual weight over the year prior to study entry as well as weight at age 20 were reported by the study participants. For current or former smokers, we collected detailed data on smoking exposure, including smoking status (never, former, or current smokers), average number of cigarettes smoked per day, age at starting and quitting, and duration of smoking. For subjects with type II diabetes, we recorded the age at diagnosis. In addition to the questionnaire survey, all consenting participants provided a 7-mL venous blood sample. Genomic DNA was extracted from peripheral lymphocytes at SRL Hachioji Laboratory and then stored at -30°C at the Department of Public Health, Aichi Medical University.

### Genotyping assays

Genotyping was performed using the Taqman SNP Genotyping Assay (Applied Biosystems, Foster City, CA, USA) at the laboratory of Aichi Cancer Center Research Institute, Nagoya, Japan. Laboratory staff were blinded to case or control status. Four quality control samples were included in each assay, and the successful genotyping rate was 100%.

### Statistical analysis

Case–control differences in selected demographic characteristics and risk factors were evaluated using t tests (for continuous variables) and Chi-square tests (for categorical variables). A chi-square test was used to test genotype frequencies in control subjects for Hardy-Weinberg equilibrium (HWE) by comparing observed genotype frequencies with those expected under HWE. A co-dominant genomic model was assumed for SNP effects. Unconditional logistic regression methods were used to estimate odds ratios (ORs) and 95% confidence intervals (CIs) for the association between rs9939609 genotypes and pancreatic cancer risk. Homozygous carriers of the common FTO rs9939609 T allele served as the reference group. All analyses were adjusted for age (continuous), sex (male or female), BMI (<20, 20–22.4, 22.5-24.9, ≥25.0), history of diabetes (yes or no), and cigarette smoking (current, former, never smokers). ORs were also estimated for the variant allele on the basis of a log-additive model. The interaction of genotype-BMI and genotype-history of diabetes with respect to pancreatic cancer risk was assessed using the likelihood ratio test. Because recent-onset diabetes may result from pancreatic cancer, we performed an analysis excluding cases who had onset of diabetes within 2 years prior to the diagnosis of pancreatic cancer.

All P-values were two-sided, with P<0.05 indicating statistical significance. All statistical analyses were performed using SAS 9.2 (SAS Institute, Inc., Cary, NC, USA).

## Results

The distribution of genotypes among control subjects did not deviate from the Hardy-Weinberg equilibrium (P=0.94). The minor allele frequency (MAF) was 0.18 among control subjects. Table 1 summarizes the characteristics of cases and controls. Both groups had a similar distribution of sex and 10-year age groups. The mean age was 65.1±8.1 years for cases, and 58.5±9.1 years for controls. Cases were more likely to be current smokers and have a history of diabetes compared with controls. Current smokers had an approximately 2.9-fold increased risk of pancreatic cancer compared with nonsmokers, after adjustment for age, sex, BMI, and history of diabetes (OR=2.86; 95%CI: 1.79-4.57). Individuals who had a BMI of 30 or more had a 1.21-fold increased risk, but the association was not statistically significant. Similar results were obtained in an additional analysis in which BMI at age 20 was used (data not shown). Risk of pancreatic cancer was significantly increased among subjects reporting a history of diabetes (OR=2.94; 95%CI: 1.90-4.57). The significant, positive association remained after excluding pancreatic cancer cases with recent-onset diabetes (OR=1.92; 95%CI: 1.20–3.08). Among control subjects, the mean BMI was 22.7±3.1 for the TT genotype, 23.2±3.3 for the TA genotype, and 21.1±2.9 for the AA genotype.

**Table 1:** Association between variations in the fat mass and obesity-associated gene and pancreatic cancer risk: a case–control study in Japan

**Characteristics**	**Case patients**	**Control subjects**	**OR (95% CI)**
	**(N=360)**	**(N=400)**	
Age group			Matching factor
<50	12 (3.3)	19 (4.8)	
50-59	44 (12.2)	79 (19.8)	
60-69	141 (39.2)	170 (42.5)	
70-79	138 (38.3)	115 (28.8)	
≥80	25 (6.9)	17 (4.3)	
Sex			Matching factor
Female	215 (59.7)	226 (56.5)	
Male	145 (40.3)	174 (43.5)	
Body mass index (kg/m^2^)			
<25	278 (77.2)	312 (78.0)	1.00
25.0-29.9	64 (17.8)	75 (18.7)	0.96 (0.65-1.43)
≥30	16 (4.4)	12 (3.0)	1.21 (0.53-2.77)
Unknown	2 (0.6)	1(0.3)	-
Smoking status			
Non-smokers	145 (40.2)	202 (50.5)	1.00
Former smokers	119 (33.1)	140 (35.0)	1.23 (0.82-1.85)
Current Smokers	96 (26.7)	58 (14.5)	2.86 (1.79-4.57)
History of diabetes			
No	269 (74.7)	362 (90.5)	1.00
Yes	87 (24.2)	35 (8.7)	2.94 (1.90-4.57)
Unknown	4 (1.1)	3 (0.8)	-

OR: odds ratio; CI: confidence interval.

OR was adjusted for sex, age, smoking status and history of diabetes.

Table 2 shows the association between variants in the FTO gene (rs9939609) and pancreatic cancer risk. Compared with individuals with the TT genotype, the multivariate adjusted OR for developing pancreatic cancer was 1.48 (95%CI: 1.07–2.04) among those with the TA genotype, and 1.66 (95%CI: 0.70–3.90) among those with the AA genotype. Under the dominant model, the OR was 1.49 (95%CI: 1.09–2.05) among carriers of the TA/AA genotype. Under the log-additive model, each additional copy of minor allele A was associated with a 1.4-fold increased risk of pancreatic cancer (OR=1.41, 95%CI: 1.07–1.85).

**Table 2:** Association between the FTO rs9939609 and pancreatic cancer risk

**FTO rs9939609**	**Cases**	**Control subjects**	**Age- and sex- adjusted OR**	**Multivariable-adjusted OR**
TT	213	271	1.00	1.00
TA	133	116	1.49 (1.09-2.03)	1.48 (1.07-2.04)
AA	14	13	1.49 (0.67-3.29)	1.66 (0.70-3.90)

OR: odds ratio ; CI: confidence interval.

Multivariable adjusted OR: adjusted for age, sex, body mass index, cigarette smoking and history of diabetes.

We found no significant interaction between FTO rs9939609 and BMI (Table 3). Individuals with both a TA/AA genotype and a history of diabetes had a 3.7-fold increased risk of pancreatic cancer compared with those with a TT genotype and no history of diabetes (Table 4), but a test for the interaction was not statistically significant.

**Table 3:** Joint associations of the FTO rs9939609 and BMI with respect to pancreatic cancer risk

**Genotype**	**BMI**	**Cases/control subjects**	**Age- and sex-adjusted OR**	**Multivariable-adjusted OR**
TT	<25	166/220	1.00	1.00
TA/AA	<25	112/92	1.69 (1.20-2.40)	1.68 (1.18-2.41)
TT	≥25	45/51	1.29 (0.81-2.04)	1.20 (0.75-1.94)
TA/AA	≥25	35/36	1.35 (0.81-2.25)	1.21 (0.71-2.07)
				P for interaction=0.29

Multivariable OR: adjusted for age, sex, cigarette smoking and history of diabetes.

**Table 4:** Joint associations of the FTO rs 9939609 and history of diabetes with respect to pancreatic cancer risk

**Genotype**	**History of diabetes**	**Cases/control subjects**	**Age- and sex-adjusted OR**	**Multivariable-adjusted OR**
TT	No	163/243	1.00	1.00
TA/AA	No	106/119	1.38 (0.99-1.93)	1.41 (1.00-1.98)
TT	Yes	34/26	1.76 (1.01-3.07)	1.70 (0.96-3.00)
TA/AA	Yes	24/8	4.03 (1.75-9.24)	3.70 (1.59-8.63)
				P for interaction=0.28

Cases were excluded if the onset of diabetes was within 2 years prior to the diagnosis of pancreatic cancer.

Multivariable OR: adjusted for age, sex, body mass index, and cigarette smoking.

## Discussion

This was a hospital-based case–control study in Japan to investigate whether genetic variations in the FTO gene were associated with pancreatic cancer risk. The main findings of our study were: 1) individuals with the FTO rs9939609 TA genotype had a significant 1.5-fold increased risk of pancreatic cancer compared with those with the TT genotype; and 2) a combination of the FTO rs9939609 TA/AA genotype and a history of diabetes significantly increased the pancreatic cancer risk, with an OR of 3.70 (95%CI: 1.59–8.63).

We found that obesity, defined as a BMI of 30 or more, was associated with 1.2-fold increased risk of pancreatic cancer, but this association was not statistically significant. In contrast to evidence of a positive association between obesity and pancreatic cancer in Western countries, available data on the role of obesity in pancreatic cancer in Japanese are inconclusive. There have been no prospective studies that have observed a clear, dose–response relation between baseline BMI and pancreatic cancer risk in the Japanese population [15,16]. Given that less than 5% of the subjects were obese in this study, it might be difficult to observe significant associations. The small percentage of obese people may be the main reason for the inconclusive results on BMI and pancreatic cancer in Asians, including Japanese [15-18]. In addition, differences in body fat distribution, in genetic predisposition to obesity and in lifestyle factors between Caucasians and Asians may contribute to the inconsistent results on BMI and pancreatic cancer risk in Asian populations [33,34].

Because of the positive association between obesity and pancreatic cancer in Caucasians and the plausible mechanisms, several research groups have hypothesized that variants in obesity-related genes might be associated with pancreatic cancer risk. The association between rs9939609 in the FTO gene was reported in one previous hospital–based case–control study conducted at the MD Anderson Cancer Center, Texas, USA [30]. Of the 15 obesity- and diabetes-associated genotypes in the FTO gene, rs9939609 was found to be positively associated with pancreatic cancer risk in persons who were overweight, whereas no increased risk was observed in persons who had a BMI of less than 25 kg/m^2^[30]. In contrast, our study showed a significant, positive association between rs9939609 TA/AA genotype and pancreatic cancer risk in individuals with a BMI of less than 25 kg/m^2^. We consider that the difference in minor allele frequency (MAF) may be the main reason, given the fact that the MAF was 18% in our study, much lower than the 38% in the MD Anderson Cancer Center case–control study. The possible differences in selection of cases and controls, patterns of linkage disequilibrium and effects of gene-gene interactions may also account for the inconsistent findings. In addition to rs9939609, rs8050136 in the FTO gene was found to be associated with pancreatic cancer risk in individuals of European ancestry [31]; however, no association was noted in another case–control study [32].

In our study, FTO rs9939609 genotypes were associated with pancreatic cancer risk. However, the mean BMI did not differ among rs9939609 genotypes for control subjects, and no significant interaction was observed between rs9939609 TA/AA genotypes and BMI with respect to pancreatic cancer risk. It is possible that the positive association observed between rs9939609 genotypes and pancreatic cancer risk may be driven by a mechanism other than adiposity. Diabetes, a well-established risk factor for pancreatic cancer, is a possible candidate. There is evidence suggesting that Asian people are more susceptible to insulin resistance at a lesser degree of obesity than Caucasians [33,34]. Besides its close association with adiposity, FTO has been shown to be associated with susceptibility to type II diabetes [21,22]. We found that individuals with a TA/AA genotype and a history of diabetes were at a 3.7-fold increased risk of pancreatic cancer. However, a test for the interaction was not statistically significant. Another possibility is that FTO is just a proxy of as yet unidentified causal variants, and it is those variants that exert their effects on rs9939609 and influence pancreatic cancer risk. Given that the function of the FTO gene is largely unknown, further studies are needed to comprehensively evaluate multiple SNPs in the FTO gene and elucidate the mechanisms by which FTO rs9939609 influences pancreatic cancer risk.

Our study has several limitations. First, it is well-known that two significant issues, namely selection bias and recall bias, plague case–control studies. Our results might have been biased if hospital controls did not represent the same population from which the cases were derived. However, the allele frequencies observed among control subjects in our study were similar to those reported in the studies of Asian populations [22]. In particular, the MAF of FTO rs9939609 was 18% in our control subjects, which is very close to that reported from a sample of 100 Japanese included in the HapMap project. Moreover, the risk estimates for current smokers and individuals with a history of diabetes were comparable to those estimated from cohort or population-based case–control studies [2,3], providing indirect evidence that selection bias might not be a serious concern in our study. Second, as for recall bias, while the analysis of the association between pancreatic cancer and BMI based on self-reported weight and height might be affected by recall bias, the association with the obesity-related genotype was not. Third, although our study included a relatively large sample size compared with previous studies conducted in Japan, the sample size may not have been large enough to detect significant gene-environment interactions in subgroups. Finally, it is possible that the results could represent a chance association and therefore replication in other independent samples is required. Despite these limitations, there are several advantages of the hospital-based design adopted in our study, including rapid case ascertainment, a high response rate from both cases and controls, and high quality genotyping.

## Conclusion

Our findings indicate that rs9939609 in the FTO gene is associated with pancreatic cancer risk in Japanese subjects, possibly through a mechanism that is independent of obesity. Because of the limited statistical power, our results need replication in other independent samples. The fast-increasing prevalence of overweight/obesity and type II diabetes in Asians provides a good opportunity to further address this association and its underlying mechanisms.

## Competing interests

The authors declare no conflict of interest.

## Authors’ contribution

SK supervised the study, SK, YL, KY designed the study, YL drafted the manuscript and conducted the statistical analysis. JU and KM performed genotyping and SNP data analysis. HI, MU, NE, HN, MM participated in data collection. All authors read and approved the final manuscript.

## Pre-publication history

The pre-publication history for this paper can be accessed here:

http://www.biomedcentral.com/1471-2407/13/337/prepub
